# Regio- and stereoselective carbometallation reactions of *N*-alkynylamides and sulfonamides

**DOI:** 10.3762/bjoc.9.57

**Published:** 2013-03-13

**Authors:** Yury Minko, Morgane Pasco, Helena Chechik, Ilan Marek

**Affiliations:** 1 The Mallat Family Organic Chemistry Laboratory, Schulich Faculty of Chemistry, and the Lise Meitner-Minerva Center for Computational Quantum Chemistry, Technion – Israel Institute of Technology, Haifa 32000, Israel

**Keywords:** carbometallation, enamides, organocopper, regiochemistry, ynamides

## Abstract

The carbocupration reactions of heterosubstituted alkynes allow the regio- and stereoselective formation of vinyl organometallic species. *N*-Alkynylamides (ynamides) are particularly useful substrates for the highly regioselective carbocupration reaction, as they lead to the stereodefined formation of vinylcopper species geminated to the amide moiety. The latter species are involved in numerous synthetically useful transformations leading to valuable building blocks in organic synthesis. Here we describe in full the results of our studies related to the carbometallation reactions of *N*-alkynylamides.

## Introduction

**Scheme 1:**
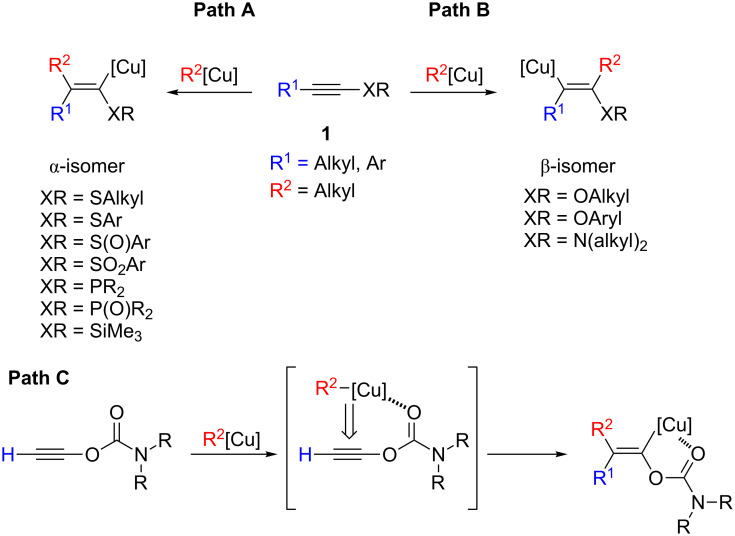
Possible regioisomers obtained in the carbocupration reaction of α-heterosubstituted acetylenes **1**.

Due to a strong differentiation of electron density on the two sp-hybridized carbon atoms, *N*-alkynylamides (ynamides) have become attractive substrates involved in many synthetically useful transformations [1–4]. The remarkable renaissance of *N*-substituted alkynes in synthesis is primarily due to the combination of the easy and straightforward access to these compounds with their notable stability [5–7]. A rather large variety of synthetic approaches have been therefore developed in the last decade that allow ynamides to be of easy access to the whole synthetic community [8–14]. Similarly, enamides are another important class of substrates involved in numerous applications in organic synthesis [5,15]. As one of the most straightforward and well-developed methods to generate polysubstituted alkenes is the carbometallation reaction of alkynes [16–18], ynamide should be a suitable substrate for the regio- and stereoselective synthesis of enamide through carbometallation reaction [19–22]. Although the stereoselectivity of the carbocupration is usually controlled through a *syn*-addition of the organocopper reagent on the triple bond [23], the regioselectivity is dependent on the nature of the α-substituent on the alkyne **1**. The presence of a donor substituent (XR = OR, NR*_2_*, **Path B**, Scheme 1) leads generally to the β-isomer in which the copper atom adds to the carbon β of the heteroatom [16–17], whereas an acceptor substituent (XR = SR, SOR, SO_2_R, SiMe_3_, PR_2_, P(O)R_2_, **Path A**, Scheme 1) would preferably give the α-isomer [16–17] (copper geminated to the heteroatom, Scheme 1). Although *N*-substituted alkynes are known to give the β-isomer, we thought that a precomplexation of the organometallic species (i.e., copper) with a polar functional group in the vicinity of the reactive center should be able to reverse the regioselectivity of the carbometallation, as we have shown for the carbocupration of ethynyl carbamate (XR = OCONR_2_, **Path C**, Scheme 1) [24].

Herein we describe our results for the regio- and stereoselective carbometallation reaction of *N-*alkynylsulfonyl amides and various *N*-alkynylcarbamates [25].

## Results and Discussion

### Carbocupration of sulfonyl-substituted ynamides

**Scheme 2:**
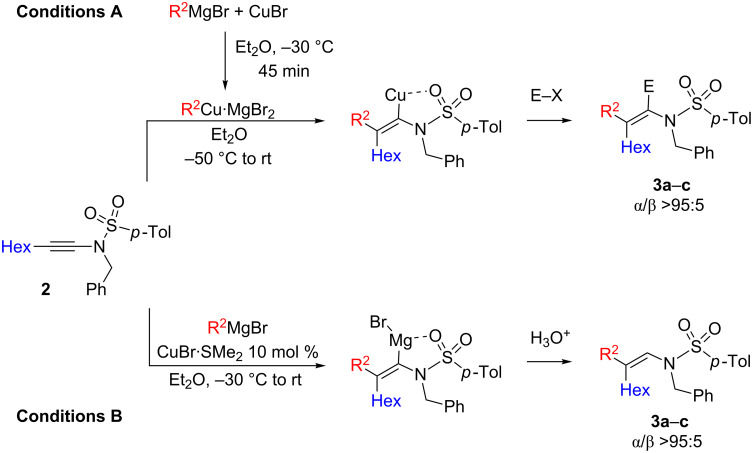
Regioselective carbometallation of *N*-alkynylsulfonamide **2**.

Our initial experiments were directed towards the carbometallation reaction of *N*-alkynylsulfonylamides, as the coordination of the organometallic species by the sulfonyl group should control the regioselectivity of the carbometallation reaction in favor of the α-isomer. When **2** was added to an organocopper (easily prepared by the addition of one equivalent of R^2^MgBr to one equivalent of CuBr in Et_2_O at −50 °C), the carbometalated products **3** were obtained in good isolated yields after hydrolysis (Scheme 2, conditions A and Table 1, entries 1–3). Some of these results were published as a preliminary note in [25].

**Table 1:** Carbometallation reactions of the *N*-alkynylsulfonamide **2**.

Entry	Conditions^a^	R^2^	E–X	Product	Yield (%)^b^

1	A	*n*-Bu	H_3_O^+^	**3a**	93
2	A	Me	H_3_O^+^	**3b**	30
3	A	*n*-Bu	AllylBr	**3c**	50
4	B	*n*-Bu	H_3_O^+^	**3a**	90
5	B	Me	H_3_O^+^	**3b**	70

^a^Conditions A: 2.0 equiv of R^2^Cu (prepared from an equimolar amount of R^2^MgBr and CuBr at −50 °C to rt in Et_2_O. Conditions B: 2.0 equiv of R^2^MgBr with 10 mol % of CuBr·Me_2_S in Et_2_O from −30 °C to rt. ^b^Yields of isolated product after flash column chromatography (based on **2**).

The reaction proceeds smoothly for classical primary alkylcopper species (Table 1, entry 1), but for groups that are known to be sluggish in carbocupration reaction, such as the introduction of a Me group (Table 1, entry 2), yield is significantly lower. The presence of the vinylcopper was proved by the reaction with allyl bromide as a classical electrophile used in organocopper chemistry to give **3c** (Table 1, entry 3). Although *N*-heterosubstituted alkynes were used in all cases, the α-isomer is regioselectively obtained, as confirmed by ^1^H NMR analysis of the crude reaction mixture after hydrolysis, through an intramolecular chelation between the *N*-sulfonamide moiety and the organocopper species. To improve the chemical yield of this transformation, we considered the copper-catalyzed carbomagnesiation reaction. However, such catalytic reaction requires a transmetallation reaction of the intermediate vinylcopper into vinylmagnesium halide, and due to the intramolecular chelation, it is usually performed at higher temperature. Thus, a thermally more stable organocopper species [formed from copper(I) bromide–dimethylsulfide complex (CuBr·Me_2_S)], was used for this copper-catalyzed carbomagnesiation reaction. We were pleased to see that the reaction is very efficient for the addition of BuMgBr, as **3a** was obtained in 90% yield as a unique α-isomer. More importantly, the copper-catalyzed methylmagnesiation now proceeded in good yield to lead to the carbometalated product **3b** in 70% yield after hydrolysis (Table 1, entry 5) [25–26].

### Carbocupration reaction of acyclic *N*-alkynylcarbamates

**Scheme 3:**
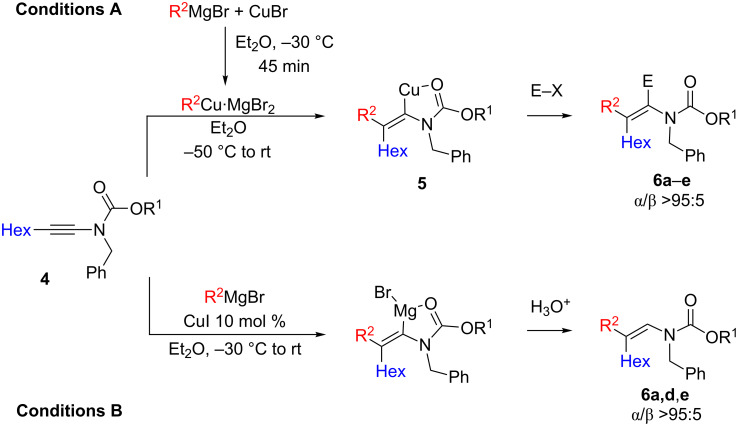
Regioselective carbometallation of ynamide **4**.

As the carbocupration of an *N*-alkynylsulfonamide could be easily achieved, we were interested to see whether such a carbocupration reaction could be extended to ynamide **4** bearing an acyclic carbamate moiety. We were pleased to find that this reaction could also be performed on *N*-alkynylcarbamate **4** with primary and secondary alkylcopper species (still obtained in diethyl ether from 1.0 equiv of a Grignard reagent and 1.0 equiv of CuBr, conditions A) to give the corresponding vinylcopper intermediate **5**. Simple hydrolysis led to the enamide **6a**,**c** in good isolated yields after purification by column chromatography (Scheme 3 and Table 2, entries 1 and 3). The vinylcopper species could also be successfully trapped at low temperature with allylbromide to give the functionalized adduct **6b** (Scheme 3, Table 2, entry 2) [25]. The addition of the Me and Ph groups proceeds more easily on the *N*-alkynylamide **4** than *N*-alkynylsulfonamide **2** as yields could reach 68 and 84%, respectively (Table 2, entries 8 and 9). Yield could be further improved when CuBr·Me_2_S was used as the copper source (Table 2, compare entries 1 and 6) [25]. In all cases, the expected α-isomer of **6** was exclusively formed and the regiochemistry was determined by NOE (nuclear Overhauser effect) between the methyl substituent and the corresponding vinylic proton (E = H, Table 2, entry 4). However, such selectivity can only be achieved in nonpolar solvent, such as Et_2_O, since the same reaction performed in THF leads mainly to the formation of the β-isomer in a α/β ratio of 18:82.

**Table 2:** Carbometallation reactions of alkynylcarbamates **4**.

Entry	Conditions^a^	R^1^	R^2^	E–X	Product	Yield (%)^b^

1	A	Et	*n*-Bu	H_3_O^+^	**6a**	72
2	A	Me	*n*-Bu	Allyl–Br	**6b**	55
3	A	Me	*c*-C_6_H_11_	H_3_O^+^	**6c**	50
4	A^c^	Et	Me	H_3_O^+^	**6d**	68
5	A^c^	Et	Ph	H_3_O^+^	**6e**	84
6	A^c^	Et	*n*-Bu	H_3_O^+^	**6a**	94
7	B	Et	*n*-Bu	H_3_O^+^	**6a**	81
8	B	Me	Me	H_3_O^+^	**6d**	60
9	B	Et	Ph	H_3_O^+^	**6e**	90
10	B	Me	*c*-C_6_H_11_	H_3_O^+^	**6c**	81

^a^Conditions A: 2.0 equiv of R^2^Cu (prepared from equimolar amount of R^2^MgBr and CuBr at −50 °C to rt in Et_2_O. Conditions B: 2.0 equiv R^2^MgBr with 10 mol % of CuI in Et_2_O from −30 °C to rt. ^b^Yields of isolated product after flash column chromatography (based on **4**). ^c^CuBr·Me_2_S complex was used as a copper source.

When *N*-alkynylamide **4** was treated under our copper-catalyzed carbomagnesiation conditions (conditions B), enamides **6** were similarly obtained as a pure α-isomer. Although this transformation requires higher temperature, yields are usually better than in reactions with the preformed organocopper species (Table 2, entries 7–10) [25].

### Carbocupration of cyclic *N*-alkynylcarbamate (chiral oxazolidinone moiety)

**Scheme 4:**
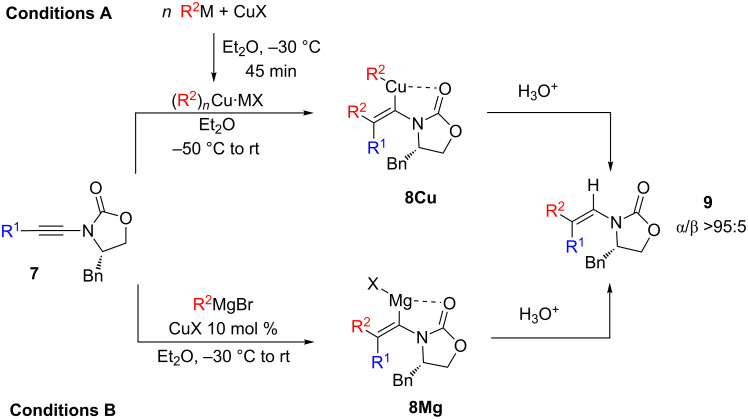
Regioselective carbometallation of cyclic *N*-alkynylcarbamate **7**.

Recently, our group was particularly interested in the carbometallation reaction of ynamides bearing chiral cyclic carbamates **7**, particularly the Evans's oxazolidinone [27], as vinylmetal species could subsequently react with a large variety of electrophiles leading to diastereomerically enriched functionalized adducts [28–30]. Thus, the carbometallation reaction of *N*-alkynylamide **7** was performed with several organometallic species as described in Scheme 4 and Table 3.

**Table 3:** Carbometallation reactions of cyclic *N*-alkynylcarbamates **7** (Scheme 4).

Entry	Conditions^a^	R^1^	R^2^	M	CuX	α/β	Product	Yield (%)^b^

1	A	Hex (**7a**)	Me	MgBr	CuI	>95:5	**9a**	75
2	B	Bu (**7b**)	Me	Li	CuI	>95:5	**9b**	59
3	B	Bu (**7b**)	Me	Li	CuBr	>95:5	**9b**	69
4	B	Bu (**7b**)	Me	Li	CuCN	90:10	**9b**	80
5	B	Bu (**7b**)	Me	Li	CuBr·Me_2_S	>95:5	**9b**	80
6	C	Bu (**7b**)	Me	MgBr	CuI	>95:5	**9b**	86
7	B	(iPr)_3_SiO(CH_2_)_2_ (**7c**)	Me	Li	CuBr·Me_2_S	>95:5	**9c**	51
8	B	Ad^1^CO_2_(CH_2_)_3_ (**7d**)	Me	Li	CuBr·Me_2_S	80:20	**9d**	90
9	B	Ph (**7e**)	Me	Li	CuBr·Me_2_S	90:10	**9e**	50
10	B	*p*-FC_6_H_4_ (**7f**)	Me	Li	CuBr·Me_2_S	>95:5	**9f**	71
11	B	*p*-MeOC_6_H_4_ (**7g**)	Me	Li	CuBr·Me_2_S	>95:5	**9g**	60

^a^Conditions A: 2.0 equiv of R^2^Cu (prepared from an equimolar amount of R^2^MgBr and CuBr at −50 °C to rt in Et_2_O. Conditions B: 2.4 equiv MeLi, 1.2 equiv CuX, Et_2_O, −30 to −20 °C, 2 h. Conditions C: 1.2 equiv MeMgBr with 10 mol % of CuI in Et_2_O from −20 to 0 °C. ^b^Yields of isolated product after flash column chromatography (based on **7**).

Our initial attempt was performed with organocopper species prepared from alkylmagnesium halide and CuBr (1:1 ratio) and ynamide **7a** (R^1^ = Hex), which cleanly led to the enamide **9a** in very good isolated yield for the addition of MeCu, and with excellent regioselectivities (α/β > 95:5, Table 3, entry 1). For subsequent reaction of **8Cu**, we were also interested in evaluating the regioselectivity of the carbometallation reaction of an organocuprate, and we were pleased to see that the same α-regioisomer was obtained. However, yields are lower with CuI and CuBr (Table 3, entries 2 and 3) than that with a more soluble complex CuBr·Me_2_S (Table 3, entry 5). However, when CuCN was employed, only an α/β ratio of 9:1 was obtained, probably due to the decreased chelating effect of the resulting mixed organometallic species (Table 3, entry 4). When the copper-catalyzed carbomagnesiation reaction was performed (Table 3, entry 6), results were very similar to those from the addition of copper or cuprate reagents (Table 3, entries 1 and 5, respectively).

**Figure 1:**
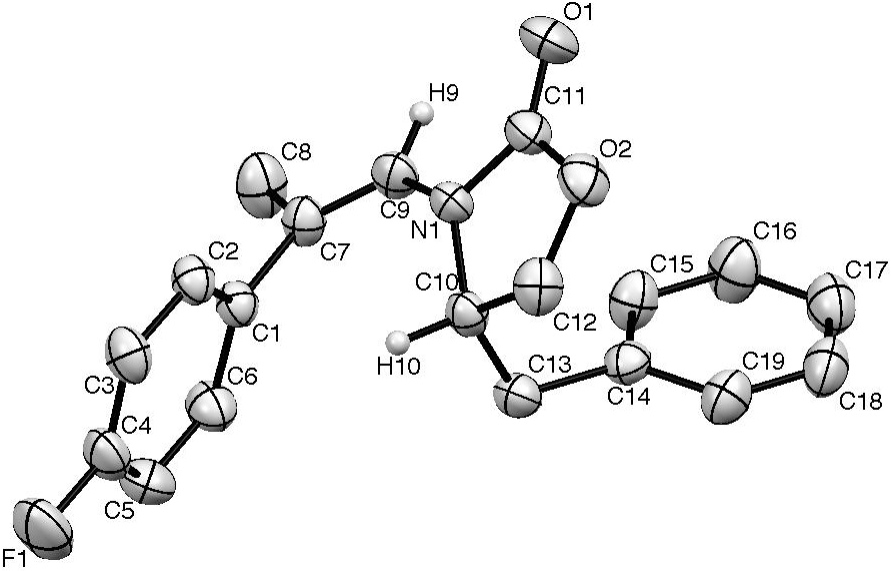
Molecular structure of **9f** (hydrogen atoms except of H9 and H10 are omitted for clarity).

Thus, the best compromise we found for the regioselective addition of a methyl group on ynamides **7a** and **7b** was through the addition of a cuprate Me_2_CuLi·Me_2_S prepared from the addition of methyllithium (2 equiv) to Me_2_S·CuBr (1 equiv). These experimental conditions were used for the stereo- and regioselective carbocupration of several functionalized ynamides **7c**–**g**. With a TIPS-protected alcohol **7c** (Table 3, entry 7), the carbocupration gave only the α-regioisomer, albeit in moderate yield. On the other hand, in the presence of the 1-adamantylester substituted substrate **7d**, the two α/β-isomers were formed in a 8:2 ratio (Table 3, entry 8). This loss of selectivity may be explained either by a competitive chelation of the carbonyl group of the ester functionality or the presence of THF (ca. 20 vol %) as a co-solvent used to dissolve the starting ynamide **7d**. When the reaction was performed with aryl-substituted ynamides (**7e**–**g**), THF had to be added as a co-solvent to improve the solubility of the starting materials as well, which may affect the resulting selectivity. Indeed, in the case of phenylsubstituted ynamine **7e**, a 1:1 mixture of Et_2_O and THF was used to avoid precipitation of the starting material, which led to a 9:1 ratio in favor of the α-isomer (Table 3, entry 9). However, for substrates bearing *p*-fluoro and *p*-methoxyphenyl substituents (**7f** and **7g** respectively), only around 8 vol % of THF was necessary to add to the reaction mixture to obtain a homogeneous solution. In such circumstances, the α-adduct was detected as the sole product of the carbocupration (Table 1, entries 10 and 11) as confirmed by NMR spectroscopy and X-ray crystallographic analysis of the major isomer of **9f** (Figure 1).

In the past few years we have witnessed the renaissance of the selective functionalization of heterosubstituted alkynes. From the pioneering work of Alexakis, Cahiez and Normant, which led to a myriad of beautiful selective transformations of alkynes, the carbocupration reaction is again at the center of interest but this time for the regioselective addition of organocopper derivatives to various ynamide species. Particularly interesting is the carbocupration of *N-*alkynyl carbamates **7**, bearing Evans’s oxazolidinone chiral auxiliary, which leads to the formation of a single regioisomer of vinylcopper **8** that may be used for the preparation of functionalized adducts in acyclic systems [28–30]. Solvent plays a detrimental role in the regioselectivity of the reaction, and only Et_2_O leads to the α-isomer, due to a precomplexation of the organocopper with the carbamate moiety.

## Supporting Information

File 1 Full experimental procedures and detailed analytical data for the synthesis of all new compounds.
