# Supplementary material for: Regio- and stereoselective carbometallation reactions of N-alkynylamides and sulfonamides
Source: Beilstein J Org Chem. 2013 Mar 13;9:526–32. doi: 10.3762/bjoc.9.57 (PMC3628878; doi:10.3762/bjoc.9.57)
Supplement: File 1 — Full experimental procedures and detailed analytical data for the synthesis of all new compounds. [file Beilstein_J_Org_Chem-09-526-s001.pdf]

Supporting Information

for

Regio- and stereoselective carbometalation reactions of *N*-alkynyl-amides and sulfonamides

Yury Minko, Morgane Pasco, Helena Chechik and Ilan Marek*

Address: The Mallat Family Organic Chemistry Laboratory, Schulich Faculty of Chemistry, and the Lise Meitner-Minerva Center for Computational Quantum Chemistry, Technion – Israel Institute of Technology, Haifa 32000, Israel

Email: Ilan Marek - chilanm@tx.technion.ac.il
*Corresponding author

Full experimental procedures and detailed analytical data for the synthesis of all new compounds

Table of contents

General Methods S2

Reagents and Materials S2

General Procedures S3

1. Stoichiometric carbocupration reaction (Method A) S3
2. Copper-catalyzed carbomagnesiation reaction (Method B) S3
3. Stoichiometric carbocupration reaction (Method C) S4

Compound characterization S4

References S18

General methods

All reactions were carried out in flame-dried glassware under a positive pressure of argon in dry solvents using standard Schlenk techniques unless otherwise indicated. The progress of the reactions was monitored by analytical TLC using glass plates precoated with silica gel with F_{254} indicator (Merck). Visualization of spots was done using UV light (254 nm), iodine, *p*-anisaldehyde, phosphomolybdic acid (PMA), and Hanessian's (cerium ammonium molybdate) stains. All organometallic compounds, dry solvents, and reagents were transferred using plastic single-use graduated syringes and oven-dried stainless-steel needles. Purification of crude mixtures was accomplished by preparative flash column chromatography on silica gel 60A (GraceResolv) using gradient mixtures of ethyl acetate–*n*-hexane. ^{1}H and ^{13}C NMR spectra were measured on a Bruker Avance 300 (300 MHz ^{1}H, 75 MHz ^{13}C) or Bruker Avance AV 400 (400 MHz ^{1}H, 100 MHz ^{13}C). Chemical shifts values (δ) are reported in ppm (calibration of spectra to the residual peak of chloroform: δ = 7.26 ppm (s) for ^{1}H NMR; δ = 77.00 ppm for ^{13}C NMR). All the ^{1}H NMR spectra are reported as follows: δ value (multiplicity, *J* coupling constant (in Hz), number of nuclei). Multiplicity abbreviations used: (s) – singlet, (d) – doublet, (dd) – doublet of doublet, (t) – triplet, (q) – quartet, (m) – multiplet, and (br) – broad signal.

Reagents and materials

All solvents were purified and dried immediately prior to use: THF and diethyl ether (HPLC grade, non-stabilized, BioLab) were dried using Innovative Technology PureSolv PS-MD-2 solvent purifier (aluminum oxide columns) and kept under a positive pressure of nitrogen. Grignard reagents were prepared from the corresponding alkyl- or arylhalides and Mg(0) in diethyl ether, transferred to a Schlenk flask, and stored at room temperature under a positive pressure of argon. The concentration of the prepared Grignard reagents was determined by back titration with a 1.00 M solution of 2-butanol in dry toluene in the presence of 2,2’-biquinoline as indicator. Methyllithium solution in diethyl ether (1.60 M) and *n*-butyllithium solution in hexane (1.60 M) were purchased from Aldrich and used as received. All starting material precursors (1-bromoalkynes) and starting chiral ynamides were prepared by literature procedures [1-4] and purified by preparative silica-gel flash column chromatography.

General procedures

1. Stoichiometric carbocupration reaction (Method A)

To a solution of copper salt (2 equiv) in dry Et_2O (10 mL) at −60 °C was added dropwise alkylmagnesium bromide (2 equiv). The reaction mixture was allowed to warm to −20 °C and stirred for 30 min. Then, the reaction mixture was cooled to −50 °C and a solution of alkynyl carbamate (1 equiv) in dry Et_2O was added dropwise. The reaction solution was allowed to warm to the indicated temperature and stirred for an additional 90 min (monitored by TLC). The reaction was quenched by a 2:1 mixture of an aqueous saturated solution of NH_4Cl and NH_4OH (30%) and allowed to warm up to room temperature. The phases were separated and the aqueous phase was extracted three times with Et_2O. The combined organic layers were washed with an aqueous saturated solution of NH_4Cl, dried over anhydrous $MgSO_4$, and concentrated under reduced pressure. Purification was accomplished by silica-gel flash column chromatography.

2. Copper-catalyzed carbomagnesiation reaction (Method B)

To a solution of copper salt (0.1 equiv) in dry Et_2O (10 mL) at –60 °C was added dropwise alkylmagnesium bromide (2 equiv). A solution of alkynyl carbamate (1 equiv) in dry Et_2O was added dropwise. The reaction solution was allowed to warm to room temperature and stirred for an additional 2 h (monitored by TLC). The reaction was quenched by a 2:1 mixture of an aqueous saturated solution of NH_4Cl and NH_4OH (30%) and allowed to warm up to room temperature. The phases were separated, and the aqueous phase was extracted three times with Et_2O. The combined organic layers were washed with an aqueous saturated solution of NH_4Cl, dried over anhydrous $MgSO_4$, and concentrated under reduced pressure. Purification was accomplished by silica-gel flash column chromatography.

3. **Stoichiometric carbocupration reaction (Method C)**

To a solution of copper salt (1.2 equiv) in dry Et_2O (8 mL) at −50 °C was added dropwise a solution of methyllithium (1.60 M in diethyl ether, 2.40 mmol, 2.4 equiv). The reaction mixture was allowed to warm to −40 °C and stirred for 30 min to form a colourless or a pale yellow solution of dimethyl cuprate–lithium bromide dimethylsulfide complex. Then, the reaction mixture was cooled to −50 °C, and a solution of alkynyl carbamate (1 equiv) in 1.5 mL of dry Et_2O was added dropwise. The reaction solution was allowed to warm to −30 °C and stirred for an additional 90 min (monitored by TLC). The reaction was quenched by a 2:1 mixture of an aqueous saturated solution of NH_4Cl and NH_4OH (30%) and allowed to warm up to room temperature. The phases were separated and the aqueous phase was extracted three times with Et_2O. The combined organic layers were washed with an aqueous saturated solution of NH_4Cl, dried over anhydrous $MgSO_4$, and concentrated under reduced pressure. Purification was accomplished by silica-gel flash column chromatography.

Compound characterization

(*Z*)-*N*-Benzyl-*N*-(2-butyloct-1-en-1-yl)-4-methylbenzenesulfonamide (3a)

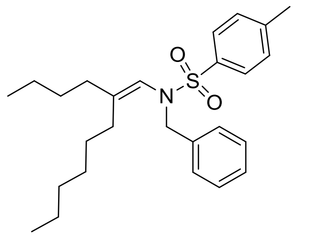

3a

The title compound was prepared using the general procedure (**Method A**) with CuI as a copper source. The carbocupration reaction of alkynyl sulfonamide **2** was performed at −20 °C. Purification by preparative column chromatography on silica gel using 5% of ethyl acetate in hexane gave the product **3a** in 93% yield (90% if **Method B** was applied). ^{1}H NMR (300 MHz, $CDCl_3$) δ 7.67 (d, *J* = 8.4 Hz, 2H); 7.31 (d, *J* = 8.4 Hz, 2H); 7.22 (m, 5H); 4.89 (s, 1H); 4.12 (s, 2H); 2.42 (s, 3H); 1.91 (t, *J* = 8.1 Hz, 2H); 1.85 (t, *J* = 8.1 Hz, 2H); 1.15 (m, 12H); 0.85 (t, *J* = 7.2 Hz, 6H). ^{13}C NMR (75 MHz, $CDCl_3$) δ 149.1; 142.8; 135.1; 134.9; 129.0; 127.7; 127.2; 127.1; 119.3; 54.5; 32.0; 31.3; 29.2; 29.1; 28.9; 26.5; 22.2; 21.6; 21.1; 13.7; 13.4 (1 signal missing due to overlap). HRMS ESI (*m*/*z*): $[MNa]^+$ calcd. for $C_{26}H_{37}NO_2SNa$ 450.2443; found 450.2437.

(Z)-*N*-Benzyl-4-methyl-*N*-(2-methyloct-1-en-1-yl)benzenesulfonamide (3b)

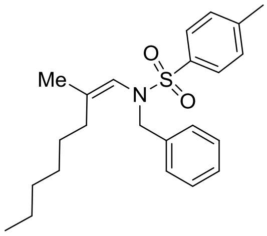

3b

The title compound was prepared using the general procedure (**Method A**) with CuI as a copper source. The carbocupration reaction of alkynyl sulfonamide **2** was performed at −20 °C. Purification by preparative column chromatography on silica gel using 5% of ethyl acetate in hexane gave the product **3b** in 30% yield. ^{1}H NMR (300 MHz, $CDCl_3$) δ 7.74 (d, *J* = 7.8 Hz, 2H); 7.31 (d, *J* = 7.8 Hz, 2H); 7.29 (m, 5H); 5.04 (s, 1H); 4.11 (s, 2H); 2.48 (s, 3H); 1.94 (t, *J* = 8.1 Hz, 2H); 1.40 (s, 3H); 1.23–1.03 (m, 8H); 0.83 (t, *J* = 7.2 Hz, 3H). ^{13}C NMR (125 MHz, $CDCl_3$) δ 146.3; 143.3; 136.2; 136.1; 135.3; 129.5; 128.2; 127.7; 124.7; 119.7; 54.9; 36.5; 31.9; 30.3; 29.7; 26.9; 22.7; 19.4; 14.1 (4 signals missing due to overlap).

(*Z*)-*N*-Benzyl-*N*-(5-butylundeca-1,4-dien-4-yl)-4-methylbenzenesulfonamide (3c)

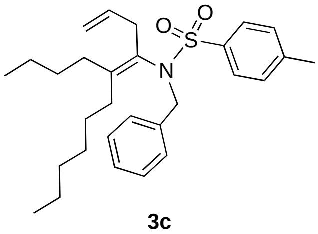

3c

The title compound was prepared using the general procedure (**Method A**) with CuI as a copper source. The carbocupration reaction of alkynyl amide **4** was performed at −20 °C. Allyl bromide (3.3 equiv) was added at −65 °C, and the reaction mixture was warmed to −30 °C and stirred for 30 min. Then the reaction was quenched and worked up as described in the general procedure. Purification by preparative column chromatography on silica gel using 5% of ethyl acetate in hexane gave the product **3c** in 50% yield. ^{1}H NMR (300 MHz, $CDCl_3$) δ 7.74 (d, *J* = 8.1 Hz, 2H); 7.29–7.21 (m, 7H); 5.63 (m, 1H); 4.92 (d, *J* = 8.1 Hz, 1H); 4.88 (d, *J* = 13.2 Hz, 1H); 4.64 (d, *J* = 14.4 Hz, 1H); 4.10 (d, *J* = 14.4 Hz, 1H); 2.84 (m, 1H); 2.82 (m, 1H); 2.42 (s, 3H); 1.87 (m, 4H); 1.23–1.02 (m, 12H); 0.86-0.81 (m, 6H). ^{13}C NMR (125 MHz, $CDCl_3$) δ 147.4; 142.6; 137.6; 136.0; 129.1; 129.0; 128.8; 127.6; 127.2; 127.1; 125.4; 115.0; 51.7; 35.3; 31.2; 30.7; 29.8; 29.3; 29.2; 27.2; 22.2; 22.2; 21.1; 13.7; 13.5 (4 signals missing due to overlap). HRMS ESI (*m*/*z*): $[MNa]^+$ calcd. for $C_{29}H_{41}NO_2SNa$ 490.2756; found 490.2750.

(*Z*)-Ethyl benzyl(2-butyloct-1-en-1-yl)carbamate (6a)

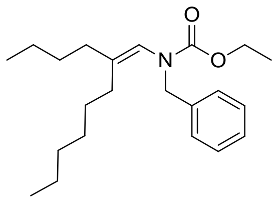

6a

The title compound was prepared using the general procedure (**Method A**) with CuBr·DMS as a copper source. The carbocupration reaction of alkynyl amide **4** was performed at −20 °C. Purification by preparative column chromatography on silica gel using 5% of ethyl acetate in hexane gave the product **6a** in 94% yield (72% when CuI was used and 81% if **Method B** was applied). ^{1}H NMR (300 MHz, $CDCl_3$) δ 7.23–7.12 (m, 5H); 5.53 (s, 1H); 4.43 (s, 2H); 4.07 (q, J^1 = 14 Hz, J^2 = 7.2 Hz, 2H); 1.89 (t, *J* = 6.6 Hz, 2H); 1.80 (m, 2H); 1.35–1.09 (m, 15H); 0.82–0.76 (m, 6H). ^{13}C NMR (75 MHz, $CDCl_3$) δ 155.3; 140.2; 136.8; 128.9; 126.2; 125.7; 120.9; 113.1; 63.5; 52.0; 31.5; 30.7; 29.3; 28.8; 28.7; 27.9; 25.8; 21.6; 13.6; 12.9 (2 signals missing due to overlap). HRMS ESI (*m*/*z*): $[MNa]^+$ calcd. for $C_{22}H_{35}NO_2Na$ 368.2565; found 368.2560.

(*Z*)-Methyl benzyl(5-butylundeca-1,4-dien-4-yl)carbamate (6b)

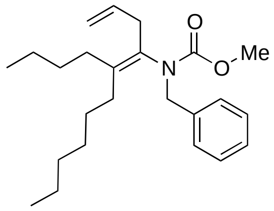

6b

The title compound was prepared using the general procedure (**Method A**) with CuBr as a copper source. The carbocupration reaction of alkynyl amide **4** was performed at −20 °C. Allyl bromide (3.3 equiv) was added at −20 °C, the reaction mixture was warmed to −15 °C and stirred for 30 min. Then the reaction was quenched and worked up as described in the general procedure. Purification by preparative column chromatography on silica gel using 1% of ethyl acetate in hexane gave the product **6b** in 55% yield. ^{1}H NMR (500 MHz, $CDCl_3$) δ 7.24 (m, 5H); 5.71 (m, 1H); 5.05 (d, *J* = 5 Hz, 1H); 5.03 (d, *J* = 11 Hz, 1H); 4.75 (d, *J* = 14 Hz, 1H); 4.46 (d, *J* = 14 Hz, 1H); 3.74 (s, 3H); 3.12 (m, 1H); 2.86 (m, 1H); 2.14 (m, 2H); 1.79 (m, 2H); 1.39–1.23 (m, 12H); 0.98 (m, 6H). ^{13}C NMR (125 MHz, $CDCl_3$) δ 158.9; 141.5; 139.7; 137.5; 131.2; 131.1; 130.4; 129.0; 117.5; 54.5; 54.2; 53.6; 37.6; 33.5; 32.8; 32.1; 31.5; 31.3; 29.0; 24.5; 24.4; 15.9 (2 signals missing due to overlap). HRMS ESI (*m*/*z*): $[MNa]^+$ calcd. for $C_{24}H_{37}NO_2Na$ 394.2722; found 394.2716.

(*E*)-Methyl benzyl(2-cyclohexyloct-1-en-1-yl)carbamate (6c)

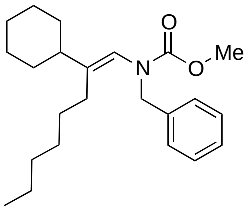

6c

The title compound was prepared using the general procedure (**Method A**) with CuI as a copper source. The carbocupration reaction of alkynyl amide **4** was performed at −20 °C. Purification by preparative column chromatography on silica gel using 5% of ethyl acetate in hexane gave the product **6c** in 50% yield (81% if **Method B** was applied). ^{1}H NMR (300 MHz, $CDCl_3$) δ 7.26–7.14 (m, 5H); 5.54 (s, 1H); 4.43 (s, 2H); 3.62 (s, 3H); 1,82–1.71 (m, 3H); 1.69–1.52 (m, 6H); 1.22–1.05 (m, 12H); 0.81 (t, *J* = 6.9 Hz, 3H). ^{13}C NMR (75 MHz, $CDCl_3$) δ 148.1; 136.7; 129.9; 127.3; 126.2; 123.7; 120.3; 52.1; 51.7; 31.4; 30.9; 29.3; 28.7; 28.3; 28.2; 26.6; 25.8; 25.3; 21.7; 21.6; 13.1 (2 signals missing due to overlap). HRMS ESI (*m*/*z*): $[MNa]^+$ calcd. for $C_{23}H_{35}NO_2Na$ 380.2565; found 380.2566.

(*Z*)-Ethyl benzyl(2-methyloct-1-en-1-yl)carbamate (6d)

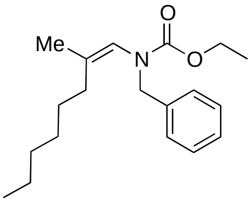

6d

The title compound was prepared using the general procedure (**Method A**) with CuI as a copper source. The carbocupration reaction of alkynyl amide **4** was performed at −20 °C. Purification by preparative column chromatography on silica gel using 5% of ethyl acetate in hexane gave the product **6d** in 68% yield (60% if **Method B** was applied). ^{1}H NMR (300 MHz, $CDCl_3$) δ 7.31–7.19 (m, 5H); 5.61 (s, 1H); 4.49 (s, 2H); 4.14 (q, J^1 = 14.1 Hz, J^2 = 6.9 Hz, 2H); 1.87 (brs, 2H); 1.61 (s, 3H); 1.26–1.06 (m, 11H); 0.86 (t, *J* = 6.6 Hz, 3H). ^{13}C NMR (75 MHz, $CDCl_3$) δ 148.1; 137.2; 127.9; 126.8; 124.3; 121.3; 113.6; 52.7; 52.3; 36.0; 31.4; 29.8; 26.3; 22.2; 18.9; 13.6; 13.4 (2 signals missing due to overlap). HRMS ESI (*m*/*z*): $[MNa]^+$ calcd. for $C_{19}H_{29}NO_2Na$ 326.2096; found 326.2110.

(*E*)-Ethyl benzyl(2-phenyloct-1-en-1-yl)carbamate (6e)

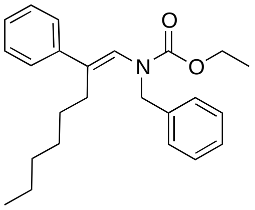

6e

The title compound was prepared using the general procedure (**Method A**) with CuI as a copper source. The carbocupration reaction of alkynyl amide **4** was performed at −20 °C. Purification by preparative column chromatography on silica gel using 5% of ethyl acetate in hexane gave the product **6e** in 84% yield (90% if **Method B** was applied). ^{1}H NMR (300 MHz, $CDCl_3$) δ 7.30–7.23 (m, 10H); 6.03 (s, 1H); 4.62 (s, 2H); 4.17 (q, J^1 = 13.8 Hz, J^2 = 6.9 Hz, 2H); 2.33–2.25 (m, 2H); 1.55–1.14 (m, 11H); 0.81 (t, *J* = 6.6 Hz, 3H). ^{13}C NMR (75 MHz, $CDCl_3$) δ 158.1; 139.5; 137.2; 128.0; 127.8; 126.0; 125.1; 124.3; 113.6; 61.3; 52.6; 36.6; 33.8; 31.5; 29.0; 26.8; 22.2; 14.2; 13.7; 13.6 (4 signals missing due to overlap). HRMS ESI (*m*/*z*): $[MNa]^+$ calcd. for $C_{24}H_{31}NO_2Na$ 388.2252; found 388.2247.

(*S*,*Z*)-4-Benzyl-3-(2-methyloct-1-en-1-yl)oxazolidin-2-one (9a)

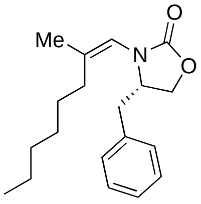

9a

The title compound was prepared using the general procedure (**Method A**) with CuI as a copper source. Purification by preparative column chromatography on silica gel using 5% of ethyl acetate in hexane gave the product **9a** in 75% yield. ^{1}H NMR (300 MHz, $CDCl_3$) δ 7.38–7.16 (m, 3H); 7.15–7.08 (m, 2H); 5.67–5.62 (m, 1H); 4.25–4.14 (m, 1H); 4.11–3.99 (m, 2H); 3.09 (dd, J^1 = 13.37 Hz, J^2 = 3.57 Hz, 1H); 2.68–2.54 (m, 1H); 2.10 (dd, J^1 = 8.48 Hz, J^2 = 6.97 Hz, 2H); 1.75 (d, *J* = 1.46 Hz, 3H); 1.53–1.37 (m, 2H); 1.36–1.23 (m, 6H); 0.87 (t, *J* = 6.9 Hz, 3H). ^{13}C NMR (100 MHz, $CDCl_3$) δ 156.7; 138.9; 135.6; 128.9; 128.8; 127.0; 116.7; 66.6; 58.9; 38.4; 31.6; 29.4; 26.9; 22.5; 19.9; 14.0 (3 signals missing due to overlap). HRMS ESI (*m*/*z*): $[MH]^+$ calcd. for $C_{19}H_{28}NO_2$ 302.2120; found 302.2129.

(*S*,*Z*)-4-Benzyl-3-(2-methylhex-1-en-1-yl)oxazolidin-2-one (9b)

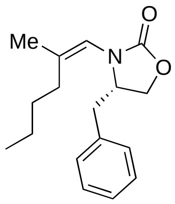

9b

The title compound was prepared using the general procedure (**Method C**) with CuBr·DMS as a copper source. Purification by preparative column chromatography on silica gel using 5% of ethyl acetate in hexane gave the product **9b** in 80% yield. ^{1}H NMR (400 MHz, $CDCl_3$) δ 7.23 (m, 3H); 7.09 (d, *J* = 7.0 Hz, 2H); 5.62 (s, 1H); 4.22–4.13 (m, 1H); 4.08–3.98 (m, 2H); 3.06 (dd, J^1 = 13.4 Hz, J^2 = 3.4 Hz, 1H); 2.59 (dd, J^1 = 13.6 Hz, J^2 = 9.1 Hz, 1H); 2.09 (dd, J^1 = 8.3, J^2 = 7.3 Hz, 2H); 1.78–1.68 (m, 3H); 1.48–1.36 (m, 2H); 1.36–1.25 (m, 2H); 0.89 (t, *J* = 7.2 Hz, 3H). ^{13}C NMR (100 MHz, $CDCl_3$) δ 156.7; 138.8; 135.5; 128.9; 128.7; 127.0; 116.7; 66.5; 58.9; 38.4; 31.3; 29.1; 22.8; 19.9; 13.9 (2 signals missing due to overlap). HRMS ESI (*m*/*z*): $[MH]^+$ calcd. for $C_{17}H_{24}NO_2$ 274.1807; found 274.1809.

(*S*,*Z*)-4-Benzyl-3-(2-methyl-5-((triisopropylsilyl)oxy)pent-1-en-1-yl)oxazolidin-2-one (9c)

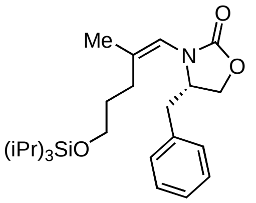

9c

The title compound was prepared using the general procedure (**Method B**) with CuBr·DMS as a copper source. Purification by preparative column chromatography on silica gel using 5% of ethyl acetate in hexane gave the product **9c** in 51% yield. ^{1}H NMR (300 MHz, $CDCl_3$) δ 7.36–7.20 (m, 3H); 7.17–7.10 (m, 2H); 5.73–5.69 (m, 1H); 4.24–4.10 (m, 2H); 4.08–4.02 (m, 1H); 3.73 (dt, J^1 = 6.2 Hz, J^2 = 1.6 Hz, 2H); 3.15–3.04 (m, 1H); 2.69–2.56 (m, 1H); 2.31–2.14 (m, 2H); 1.79 (d, *J* = 1.4 Hz, 3H); 1.77–1.65 (m, 2H); 1.20–0.92 (m, 21H). ^{13}C NMR (75 MHz, $CDCl_3$) δ 156.7; 137.7; 135.5; 128.9; 128.7; 127.0; 116.9; 66.5; 63.1; 58.6; 38.2; 30.4; 28.2; 20.1; 17.9; 11.8 (9 signals missing due to overlap). HRMS ESI (*m*/*z*): $[MNa]^+$ calcd. for $C_{25}H_{42}NO_3Si$ 432.2934; found 432.2933.

(3*S*,5*S*,7*S*)-(*Z*)-5-((*S*)-4-Benzyl-2-oxooxazolidin-3-yl)-4-methylpent-4-en-1-yl adamantane-1-carboxylate (9d)

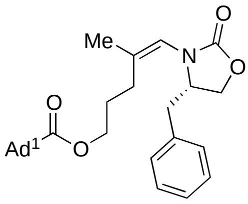

9d

The title compound was prepared using the general procedure (**Method B**) using CuBr·DMS as a copper source. Purification by preparative column chromatography on silica gel using 5% of ethyl acetate in hexane gave the product **9d** in 90% yield. ^{1}H NMR (300 MHz, $CDCl_3$) δ 7.34–7.17 (m, 3H); 7.15–7.07 (m, 2H); 5.64 (d, *J* = 1.1 Hz, 1H); 4.27–3.98 (m, 5H); 3.07 (dd, J^1 = 13.4 Hz, J^2 = 3.7 Hz, 1H); 2.68–2.53 (m, 1H); 2.23–2.12 (m, 2H); 1.96 (s, 3H); 1.76 (m, 18H). ^{13}C NMR (100 MHz, $CDCl_3$) δ 177.5; 156.6; 137.6; 135.4; 129.0; 128.9; 128.8; 128.8; 127.1; 117.6; 66.7; 63.8; 58.9; 40.6; 38.8; 36.4; 28.3; 27.9; 26.2; 19.9 (7 signals missing due to overlap). HRMS ESI (*m*/*z*): $[MNa]^+$ calcd. for $C_{27}H_{35}NO_4Na$ 460.2464; found 460.2487.

(*S*,*Z*)-4-Benzyl-3-(2-phenylprop-1-en-1-yl)oxazolidin-2-one (9e)

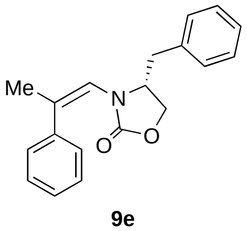

9e

The title compound was prepared using the general procedure (**Method B**) with CuBr·DMS as a copper source. A 1:1 mixture of Et_2O and THF was used to avoid precipitation of the starting material. Purification by preparative column chromatography on silica gel using 5% of ethyl acetate in hexane gave the product **9e** in 50% yield. ^{1}H NMR (300 MHz, $CDCl_3$) δ 7.47–7.33 (m, 5H); 7.20–7.13 (m, 3H); 6.64-6.58 (m, 2H); 6.49–6.47 (m, 1H); 3.96–3.89 (m, 2H); 3.71–3.60 (m, 1H); 2.84 (dd, J^1 = 13.32 Hz, J^2 = 3.59 Hz, 1H); 2.28 (dd, J^1 = 13.31 Hz, J^2 = 10.27 Hz, 1H); 2.14 (d, *J* = 1.43 Hz, 3H). ^{13}C NMR (75 MHz, $CDCl_3$) δ 156.7; 139.7; 135.3; 128.8; 128.4; 128.0; 127.6; 126.7; 125.1; 118.4; 65.7; 55.5; 36.8; 23.0 (5 signals missing due to overlap). HRMS ESI (*m*/*z*): $[MNa]^+$ calcd. for $C_{19}H_{20}NO_2$ 294.1494; found 294.1498.

(*S*,*Z*)-4-Benzyl-3-(2-(4-fluorophenyl)prop-1-en-1-yl)oxazolidin-2-one (9f)

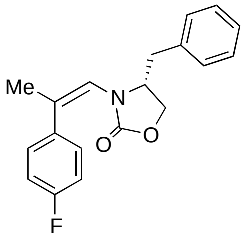

9f

The title compound was prepared using the general procedure (**Method B**) with CuBr·DMS as a copper source. A 12:1 mixture of Et_2O and THF was used to avoid precipitation of the starting material. Purification by preparative column chromatography on silica gel using 5% of ethyl acetate in hexane gave the product **9f** in 71% yield. Single crystals suitable for X-ray crystallographic analysis were obtained by slow evaporation of a saturated solution of the title compound in $CHCl_3$ at room temperature. ^{1}H NMR (300 MHz, $CDCl_3$) δ 7.39–7.28 (m, 2H); 7.24–7.06 (m, 5H); 6.68 (dd, J^1 = 7.3 Hz, J^2 = 2.1 Hz, 2H); 6.46–6.43 (m, 1H); 3.98–3.91 (m, 2H); 3.70–3.57 (m, 1H); 2.82 (dd, J^1 = 13.4 Hz, J^2 = 3.7 Hz, 1H); 2.32 (dd, J^1 = 13.4 Hz, J^2 = 10.0 Hz, 1H); 2.09 (t, J = 1.4 Hz, 3H). ^{13}C NMR (75 MHz, $CDCl_3$) δ 163.6; 160.3; 156.7; 135.6; 135.6; 135.2; 129.7; 129.6; 128.8; 128.7; 128.6; 128.6; 126.9; 124.9; 118.9; 115.6; 115.3; 65.8; 55.6; 36.9; 22.9. HRMS ESI (*m*/*z*): $[MNa]^+$ calcd. for $C_{19}H_{19}FNO_2$ 312.1400; found 312.1404.

(*S*,*Z*)-4-Benzyl-3-(2-(4-methoxyphenyl)prop-1-en-1-yl)oxazolidin-2-one (9g)

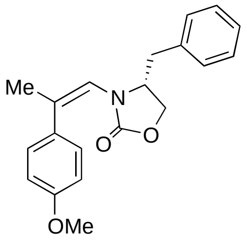

9g

The title compound was prepared using the general procedure (**Method B**) with CuBr·DMS as a copper source. A 12:1 mixture of Et_2O and THF was used to avoid precipitation of the starting material. Purification by preparative column chromatography on silica gel using 5% of ethyl acetate in hexane gave the product **9g** in 60% yield. ^{1}H NMR (300 MHz, $CDCl_3$) δ 7.32–7.25 (m, 2H); 7.22–7.13 (m, 3H); 6.98–6.92 (m, 2H); 6.72–6.63 (m, 2H); 6.42–6.39 (m, 1H); 4.01–3.89 (m, 2H); 3.85 (s, 3H); 3.69 (ddt, J^1 = 10.9 Hz, J^2 = 7.3 Hz, J^3 = 3.7 Hz, 1H); 2.83 (dd, J^1 = 13.4 Hz, J^2 = 3.6 Hz, 1H); 2.30 (dd, J^1 = 13.4 Hz, J^2 = 10.0 Hz, 1H); 2.11 (d, J = 1.4 Hz, 3H). ^{13}C NMR (75 MHz, $CDCl_3$) δ 159.0; 156.8; 135.4; 131.8; 129.1; 128.9; 128.5; 126.7;

125.4; 117.9; 113.8; 65.9; 55.6; 55.2; 36.9; 22.9 (4 signals missing due to overlap). HRMS ESI (*m/z*): $[MNa]^+$ calcd. for $C_{20}H_{22}NO_3$ 324.1600; found 324.1598.

X-ray data for compound 9f

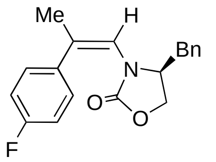

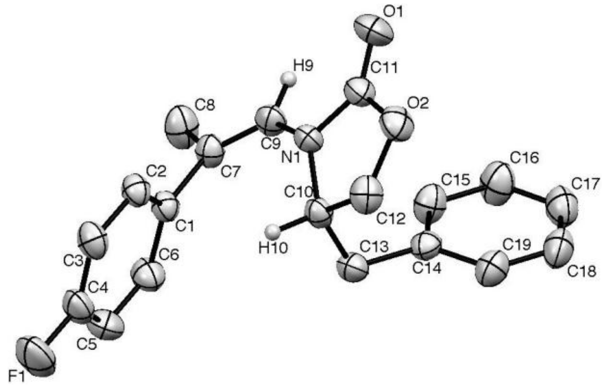

Figure S1: Crystal structure of compound **9f** (ORTEP plot, ellipsoid probability 25%).

Table 1. Crystal data and structure refinement for **9f**.

Empirical formula	C19 H19 F N O2
Formula weight	312.35
Temperature	296(2) K
Wavelength	0.71073 Å
Crystal system, space group	Tetragonal, P 43 21 2
Unit cell dimensions	a = 8.4073(7) Å alpha = 90 deg.<br>b = 8.4073(7) Å beta = 90 deg.<br>c = 46.352(3) Å gamma = 90 deg.
Volume	3276.3(5) $Å^3$
Z, Calculated density	8, 1.266 Mg/m^3
Absorption coefficient	0.089 mm^{-1}
F(000)	1320
Crystal size	0.506 x 0.302 x 0.268 mm
Theta range for data collection	2.46 to 24.50 deg.
Limiting indices	-9<=h<=3, -9<=k<=4, -25<=l<=52
Reflections collected / unique	4057 / 2637 [R(int) = 0.0218]

Completeness to theta = 24.50	96.8 %
Absorption correction	Semi-empirical from equivalents
Max. and min. transmission	0.9764 and 0.9561
Refinement method	Full-matrix least-squares on F^2
Data / restraints / parameters	2637 / 0 / 209
Goodness-of-fit on F^2	1.012
Final R indices [I>2sigma(I)]	R1 = 0.0481, wR2 = 0.1338
R indices (all data)	R1 = 0.0679, wR2 = 0.1480
Absolute structure parameter	2(2)
Extinction coefficient	0.032(3)
Largest diff. peak and hole	0.145 and -0.146 e.Å $^{-3}$

Atomic coordinates (x 10^4) and equivalent isotropic displacement parameters (Å^2 x 10^3) for 9f. U(eq) is defined as one third of the trace of the orthogonalized Uij tensor.

	x	y	z	U(eq)
N(1)	8869(3)	1296(2)	5367(1)	59(1)
F(1)	9281(3)	-4842(3)	6186(1)	130(1)
O(1)	9545(3)	2888(3)	4983(1)	112(1)
O(2)	7451(3)	1270(3)	4962(1)	86(1)
C(1)	10150(3)	-291(3)	5906(1)	60(1)
C(2)	10531(4)	-1590(3)	5736(1)	65(1)
C(3)	10236(4)	-3130(3)	5827(1)	79(1)
C(4)	9577(4)	-3336(4)	6094(1)	83(1)
C(5)	9189(5)	-2104(5)	6268(1)	92(1)
C(6)	9494(4)	-586(4)	6177(1)	81(1)
C(7)	10516(3)	1336(3)	5806(1)	64(1)
C(8)	11611(5)	2301(4)	5994(1)	96(1)
C(9)	9979(3)	1945(3)	5559(1)	69(1)
C(10)	7452(3)	362(3)	5443(1)	61(1)
C(11)	8723(4)	1903(4)	5097(1)	72(1)
C(12)	6751(4)	100(4)	5144(1)	80(1)
C(13)	6356(4)	1188(4)	5656(1)	70(1)
C(14)	5758(3)	2795(4)	5565(1)	67(1)
C(15)	6600(4)	4139(4)	5627(1)	85(1)
C(16)	6061(5)	5637(4)	5545(1)	105(1)
C(17)	4632(5)	5799(5)	5408(1)	99(1)
C(18)	3774(4)	4464(5)	5346(1)	94(1)
C(19)	4305(4)	2989(4)	5425(1)	86(1)

Bond lengths [Å] and angles [deg] for 9f.

N(1)-C(11)	1.360(3)
N(1)-C(9)	1.400(4)
N(1)-C(10)	1.469(4)
F(1)-C(4)	1.359(4)
O(1)-C(11)	1.199(4)
O(2)-C(11)	1.347(4)
O(2)-C(12)	1.421(4)
C(1)-C(2)	1.384(4)
C(1)-C(6)	1.394(4)
C(1)-C(7)	1.476(4)
C(2)-C(3)	1.383(4)
C(2)-H(2)	0.9601
C(3)-C(4)	1.366(5)
C(3)-H(3)	0.9599
C(4)-C(5)	1.354(5)
C(5)-C(6)	1.369(5)
C(5)-H(5)	0.9600
C(6)-H(6)	0.9596
C(7)-C(9)	1.331(4)
C(7)-C(8)	1.504(4)
C(8)-H(8A)	0.9599
C(8)-H(8B)	0.9600
C(8)-H(8C)	0.9604
C(9)-H(9)	0.9600
C(10)-C(13)	1.518(4)
C(10)-C(12)	1.522(4)
C(10)-H(10)	0.9600
C(12)-H(12A)	0.9600
C(12)-H(12B)	0.9600
C(13)-C(14)	1.501(4)
C(13)-H(13A)	0.9600
C(13)-H(13B)	0.9605
C(14)-C(15)	1.363(5)
C(14)-C(19)	1.393(4)
C(15)-C(16)	1.390(5)
C(15)-H(15)	0.9601
C(16)-C(17)	1.367(5)
C(16)-H(16)	0.9600
C(17)-C(18)	1.365(5)
C(17)-H(17)	0.9601
C(18)-C(19)	1.368(5)
C(18)-H(18)	0.9599
C(19)-H(19)	0.9600
C(11)-N(1)-C(9)	120.0(3)
C(11)-N(1)-C(10)	110.3(2)
C(9)-N(1)-C(10)	126.8(2)
C(11)-O(2)-C(12)	109.2(2)

C(2)-C(1)-C(6)	117.6(3)
C(2)-C(1)-C(7)	120.3(2)
C(6)-C(1)-C(7)	122.0(3)
C(3)-C(2)-C(1)	121.7(3)
C(3)-C(2)-H(2)	118.1
C(1)-C(2)-H(2)	120.1
C(4)-C(3)-C(2)	117.8(3)
C(4)-C(3)-H(3)	121.5
C(2)-C(3)-H(3)	120.4
C(5)-C(4)-F(1)	118.6(3)
C(5)-C(4)-C(3)	122.8(3)
F(1)-C(4)-C(3)	118.5(3)
C(4)-C(5)-C(6)	118.9(3)
C(4)-C(5)-H(5)	118.4
C(6)-C(5)-H(5)	122.8
C(5)-C(6)-C(1)	121.3(3)
C(5)-C(6)-H(6)	119.9
C(1)-C(6)-H(6)	118.7
C(9)-C(7)-C(1)	123.7(2)
C(9)-C(7)-C(8)	119.8(3)
C(1)-C(7)-C(8)	116.4(3)
C(7)-C(8)-H(8A)	107.7
C(7)-C(8)-H(8B)	109.9
H(8A)-C(8)-H(8B)	109.5
C(7)-C(8)-H(8C)	110.8
H(8A)-C(8)-H(8C)	109.5
H(8B)-C(8)-H(8C)	109.5
C(7)-C(9)-N(1)	128.4(3)
C(7)-C(9)-H(9)	113.6
N(1)-C(9)-H(9)	117.9
N(1)-C(10)-C(13)	113.7(2)
N(1)-C(10)-C(12)	100.1(2)
C(13)-C(10)-C(12)	115.1(3)
N(1)-C(10)-H(10)	110.2
C(13)-C(10)-H(10)	108.8
C(12)-C(10)-H(10)	108.7
O(1)-C(11)-O(2)	121.8(3)
O(1)-C(11)-N(1)	127.7(3)
O(2)-C(11)-N(1)	110.4(3)
O(2)-C(12)-C(10)	106.1(2)
O(2)-C(12)-H(12A)	111.1
C(10)-C(12)-H(12A)	111.7
O(2)-C(12)-H(12B)	107.9
C(10)-C(12)-H(12B)	110.5
H(12A)-C(12)-H(12B)	109.5
C(14)-C(13)-C(10)	115.7(2)
C(14)-C(13)-H(13A)	106.5
C(10)-C(13)-H(13A)	110.3
C(14)-C(13)-H(13B)	105.4
C(10)-C(13)-H(13B)	109.2
H(13A)-C(13)-H(13B)	109.4
C(15)-C(14)-C(19)	117.1(3)
C(15)-C(14)-C(13)	120.9(3)
C(19)-C(14)-C(13)	121.9(3)
C(14)-C(15)-C(16)	121.7(3)
C(14)-C(15)-H(15)	117.2
C(16)-C(15)-H(15)	121.1
C(17)-C(16)-C(15)	120.2(4)

C(17)-C(16)-H(16)	117.1
C(15)-C(16)-H(16)	122.7
C(16)-C(17)-C(18)	118.7(4)
C(16)-C(17)-H(17)	123.0
C(18)-C(17)-H(17)	118.0
C(17)-C(18)-C(19)	121.1(3)
C(17)-C(18)-H(18)	118.1
C(19)-C(18)-H(18)	120.5
C(18)-C(19)-C(14)	121.2(3)
C(18)-C(19)-H(19)	117.3
C(14)-C(19)-H(19)	121.3

Symmetry transformations used to generate equivalent atoms:

Anisotropic displacement parameters (Å^2 x 10^3) for 9f.
The anisotropic displacement factor exponent takes the form:
$-2 pi^2$ [$h^2 a^{*2}$ U11 + ... + 2 h k a* b* U12]

	U11	U22	U33	U23	U13	U12
N(1)	62(1)	55(1)	60(1)	11(1)	1(1)	-2(1)
F(1)	145(2)	88(2)	157(2)	51(2)	-14(2)	-24(1)
O(1)	110(2)	125(2)	101(2)	52(2)	18(2)	-9(2)
O(2)	97(2)	102(2)	60(1)	6(1)	-5(1)	9(1)
C(1)	66(2)	60(2)	54(1)	2(1)	-3(1)	5(1)
C(2)	75(2)	60(2)	59(2)	-1(1)	-1(1)	10(1)
C(3)	99(2)	53(2)	84(2)	1(2)	-8(2)	9(2)
C(4)	92(2)	61(2)	97(2)	23(2)	-9(2)	-4(2)
C(5)	94(3)	105(3)	77(2)	23(2)	16(2)	-1(2)
C(6)	95(2)	78(2)	69(2)	3(2)	12(2)	14(2)
C(7)	66(2)	53(2)	74(2)	-2(1)	-10(1)	0(1)
C(8)	104(3)	76(2)	107(3)	-3(2)	-37(2)	-7(2)
C(9)	68(2)	51(2)	88(2)	8(1)	-5(2)	-8(1)
C(10)	66(2)	50(1)	67(2)	7(1)	-4(1)	-4(1)
C(11)	77(2)	76(2)	64(2)	15(2)	7(2)	6(2)
C(12)	85(2)	75(2)	80(2)	-10(2)	-12(2)	6(2)
C(13)	72(2)	72(2)	67(2)	10(2)	5(2)	-5(2)
C(14)	61(2)	77(2)	62(2)	-1(1)	9(1)	5(1)
C(15)	80(2)	76(2)	99(2)	-14(2)	-18(2)	5(2)
C(16)	101(3)	68(2)	146(4)	-9(2)	-15(3)	7(2)
C(17)	110(3)	86(3)	100(3)	7(2)	-3(2)	27(2)
C(18)	79(2)	106(3)	96(2)	-1(2)	-10(2)	29(2)
C(19)	72(2)	96(2)	91(2)	-5(2)	-8(2)	6(2)

Hydrogen coordinates (x 10^4) and isotropic displacement parameters (Å^2 x 10^3) for 9f.

	x	y	z	U(eq)
H(2)	10982	-1438	5548	78
H(3)	10588	-4019	5713	95
H(5)	8730	-2336	6453	110
H(6)	9329	293	6306	97
H(8A)	11739	3325	5905	115
H(8B)	12627	1786	6008	115
H(8C)	11169	2430	6183	115
H(9)	10468	2938	5508	83
H(10)	7759	-650	5521	73
H(12A)	5613	191	5145	96
H(12B)	7049	-927	5071	96
H(13A)	5433	546	5692	84
H(13B)	6917	1362	5833	84
H(15)	7570	4014	5734	102
H(16)	6656	6591	5581	126
H(17)	4249	6798	5336	119
H(18)	2740	4593	5261	112
H(19)	3683	2089	5366	103

Torsion angles [deg] for 9f.

C(6)-C(1)-C(2)-C(3)	1.8(4)
C(7)-C(1)-C(2)-C(3)	178.5(3)
C(1)-C(2)-C(3)-C(4)	-1.2(5)
C(2)-C(3)-C(4)-C(5)	0.9(6)
C(2)-C(3)-C(4)-F(1)	-179.9(3)
F(1)-C(4)-C(5)-C(6)	179.6(3)
C(3)-C(4)-C(5)-C(6)	-1.2(6)
C(4)-C(5)-C(6)-C(1)	1.9(6)
C(2)-C(1)-C(6)-C(5)	-2.1(5)
C(7)-C(1)-C(6)-C(5)	-178.8(3)
C(2)-C(1)-C(7)-C(9)	57.5(4)
C(6)-C(1)-C(7)-C(9)	-125.9(3)
C(2)-C(1)-C(7)-C(8)	-120.1(3)
C(6)-C(1)-C(7)-C(8)	56.4(4)
C(1)-C(7)-C(9)-N(1)	7.7(5)
C(8)-C(7)-C(9)-N(1)	-174.7(3)
C(11)-N(1)-C(9)-C(7)	-165.3(3)
C(10)-N(1)-C(9)-C(7)	36.3(5)
C(11)-N(1)-C(10)-C(13)	-105.8(3)
C(9)-N(1)-C(10)-C(13)	54.3(3)
C(11)-N(1)-C(10)-C(12)	17.4(3)
C(9)-N(1)-C(10)-C(12)	177.6(3)
C(12)-O(2)-C(11)-O(1)	177.7(3)
C(12)-O(2)-C(11)-N(1)	-3.8(3)
C(9)-N(1)-C(11)-O(1)	7.1(5)
C(10)-N(1)-C(11)-O(1)	168.8(3)
C(9)-N(1)-C(11)-O(2)	-171.3(2)
C(10)-N(1)-C(11)-O(2)	-9.6(3)
C(11)-O(2)-C(12)-C(10)	14.9(3)
N(1)-C(10)-C(12)-O(2)	-18.9(3)

C(13)-C(10)-C(12)-O(2)	103.3(3)
N(1)-C(10)-C(13)-C(14)	57.0(3)
C(12)-C(10)-C(13)-C(14)	-57.6(3)
C(10)-C(13)-C(14)-C(15)	-88.3(4)
C(10)-C(13)-C(14)-C(19)	94.0(4)
C(19)-C(14)-C(15)-C(16)	-1.9(5)
C(13)-C(14)-C(15)-C(16)	-179.8(3)
C(14)-C(15)-C(16)-C(17)	1.9(7)
C(15)-C(16)-C(17)-C(18)	-1.6(7)
C(16)-C(17)-C(18)-C(19)	1.5(6)
C(17)-C(18)-C(19)-C(14)	-1.6(6)
C(15)-C(14)-C(19)-C(18)	1.8(5)
C(13)-C(14)-C(19)-C(18)	179.6(3)

References

1. Zhang, Y.; Hsung, R. P.; Tracey, M. R.; Kurtz, K. C. M.; Vera, E. L. *Org. Lett.* **2004,** *6,* 1151–1154. doi:10.1021/ol049827e
2. Yao, P.-Y.; Zhang, Y.; Hsung, R. P.; Zhao, K. A. *Org. Lett.* **2008,** *10,* 4275–4278. doi:10.1021/ol801711p
3. Das, J. P.; Chechik, H.; Marek, I. *Nat. Chem.* **2009,** *1,* 128–132. doi:10.1038/nchem.131
4. Minko, Y.; Pasco, M.; Lercher, L.; Botoshansky, M.; Marek, I. *Nature* **2012,** *490,* 522–526. doi:10.1038/nature11569
